# *tidy tree*: A New Layout for Phylogenetic Trees

**DOI:** 10.1093/molbev/msac204

**Published:** 2022-09-26

**Authors:** Simon Penel, Damien M de Vienne

**Affiliations:** Univ Lyon, Université Lyon 1, CNRS, Laboratoire de Biométrie et Biologie Évolutive UMR5558, F-69622 Villeurbanne, France; Univ Lyon, Université Lyon 1, CNRS, Laboratoire de Biométrie et Biologie Évolutive UMR5558, F-69622 Villeurbanne, France

**Keywords:** tree layout, visualization, phylogram

## Abstract

Many layouts exist for visualizing phylogenetic trees, allowing to display the same information (evolutionary relationships) in different ways. For large phylogenies, the choice of the layout is a key element, because the printable area is limited, and because interactive on-screen visualizers can lead to unreadable phylogenetic relationships at high zoom levels. A visual inspection of available layouts for rooted trees reveals large empty areas that one may want to fill in order to use less drawing space and eventually gain readability. This can be achieved by using the nonlayered tidy tree layout algorithm that was proposed earlier but was never used in a phylogenetic context so far. Here, we present its implementation, and we demonstrate its advantages on simulated and biological data (the measles virus phylogeny). Our results call for the integration of this new layout in phylogenetic software. We implemented the nonlayered tidy tree layout in R language as a stand-alone function (available at https://github.com/damiendevienne/non-layered-tidy-trees), as an option in the tree plotting function of the R package *ape*, and in the recent tool for visualizing reconciled phylogenetic trees *thirdkind* (https://github.com/simonpenel/thirdkind/wiki).

## Introduction

A phylogenetic tree is a visual representation of the evolutionary descent of different species, organisms, or genes from a common ancestor (Baum 2008). This diagrammatic representation of evolution is a central object in biology and beyond, used on a daily basis not only in laboratories but also in mainstream media for communication purposes.

**Fig. 1.**
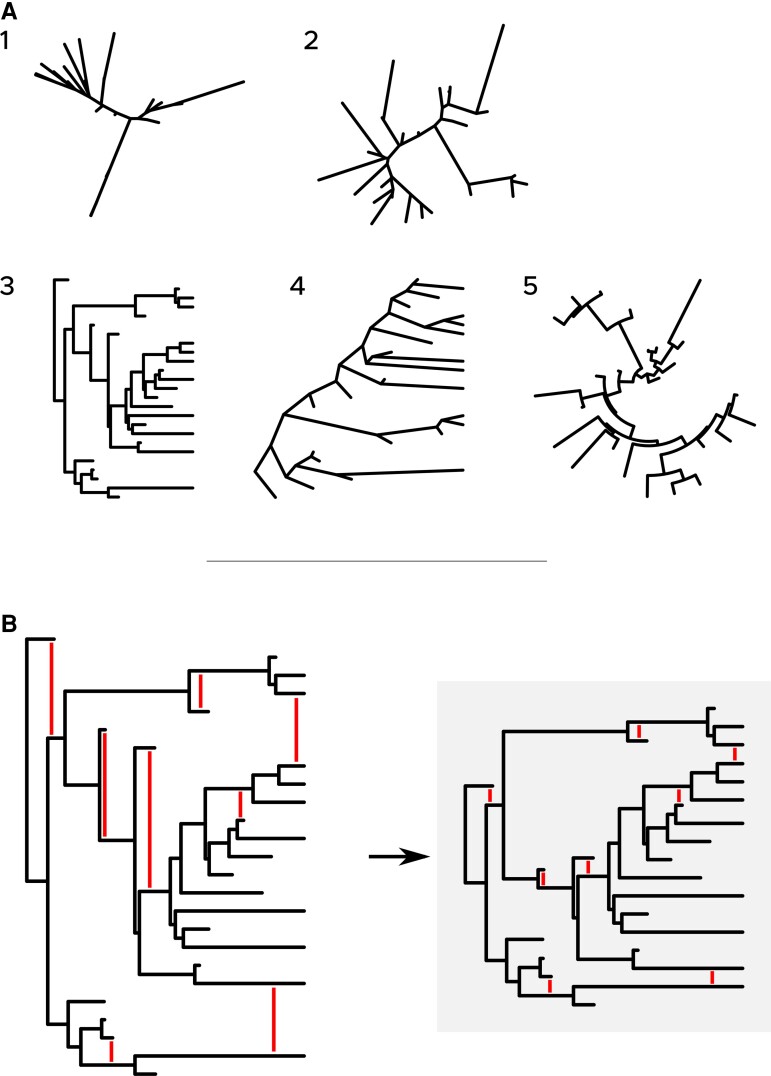
Illustration of current and proposed layouts for phylogenetic tree representation. (*A*) Five classical layouts for representing phylogenetic trees. Unrooted: equal-angle (1), daylight (2); rooted: phylogram-rectangular (3), phylogram-slanted (4), and circular (5). (*B*) New layout: the nonlayered tidy tree. It is obtained by compressing vertically the rectangular phylogram layout as indicated by the vertical red lines in both trees.

Multiple layouts exist for representing phylogenetic trees (fig. 1*A*), the choice of which depends on the nature of the tree (rooted or unrooted) and on graphical and esthetical choices made by authors to ensure that the tree displayed best represents what needs to be. A wide variety of different phylogeny plotting layouts were already in broad use when Felsenstein described them in his book “Inferring Phylogenies” (2003, chapter 34), and in the intervening two decades, relatively few additional plotting styles have been described or adopted.

**Fig. 2.**
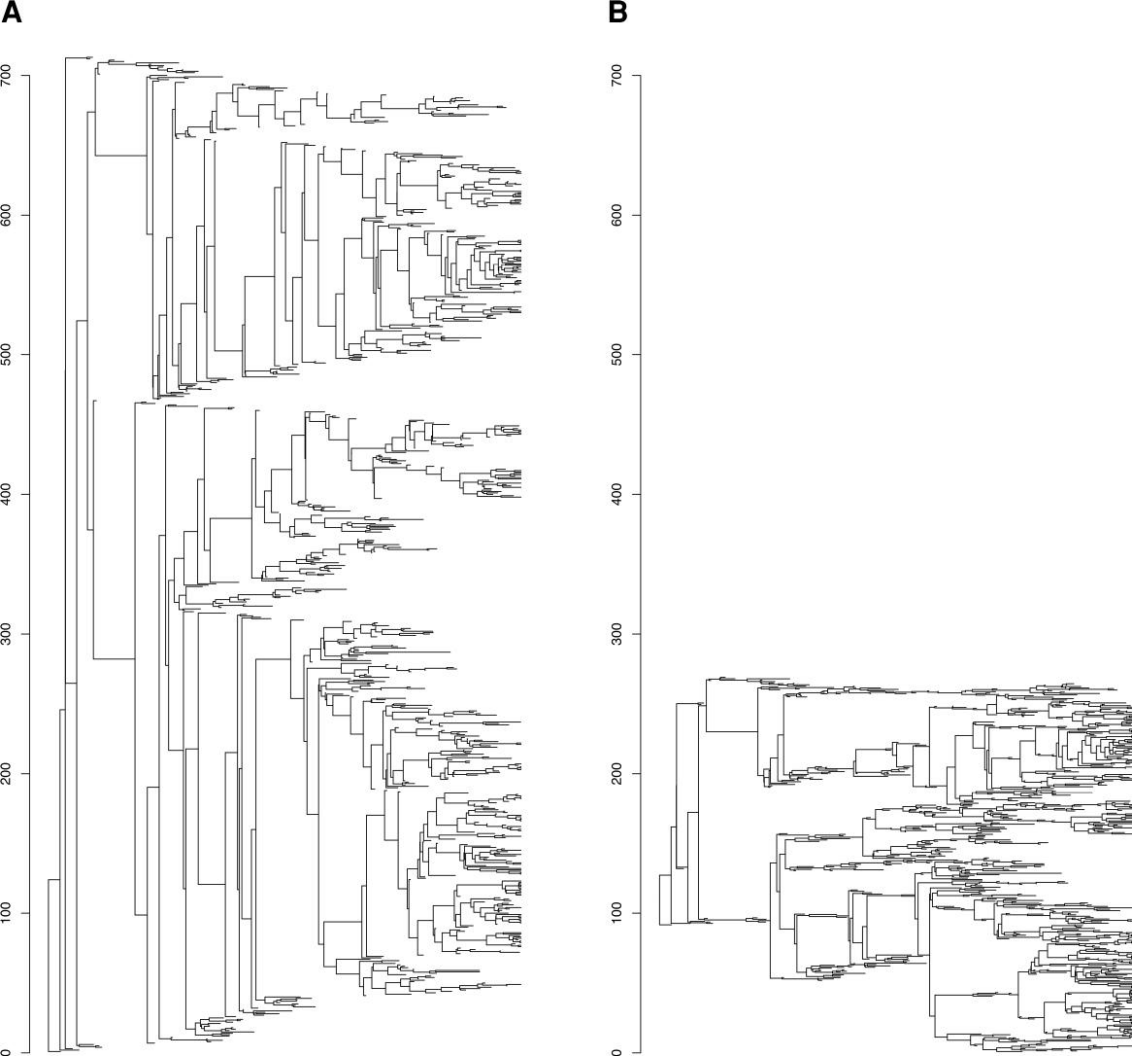
Illustration of the compression achieved by the nonlayered tidy tree algorithm. A random tree was plotted with the classical phylogram layout (*A*) and with the new nonlayered tidy tree layout (*B*), keeping the same scale for the axes. Note that the minimum possible distance between two contemporaneous branches is the same on the two layouts presented here.

**Fig. 3.**
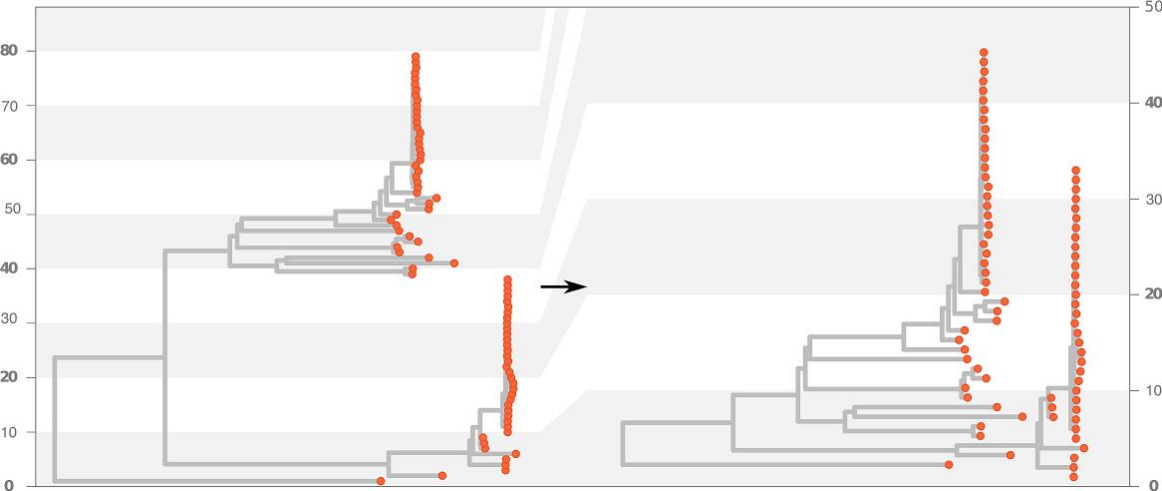
Illustration of the use of the nonlayered tidy tree layout on a phylogeny of the measles virus from North America. Left: before applying the nonlayered tidy tree algorithm. Right: after having applied the algorithm. We notice the change of the *y*-axis between the two plots, highlighted by the background gray strips.

For rooted phylogenies, phylogram and circular layouts (fig. 1*A*-3–5) are the most popular ones. In these representations, the tips of the tree are equally distributed, along a line (rectangular layout) or along a circle (circular layout). In the common case where the tree is not ultrametric (all tips do not align vertically, as in fig. 1*A-*3), these layouts may leave large empty areas. To date, and unlike for the unrooted case with the equal daylight layout (fig. 1*A-*2), no layout was made accessible to phylogeneticists to make a better use of these empty areas. Such a layout, however, exists: the nonlayered tidy tree layout, which can be drawn in linear time following the Reingold–Tilford algorithm(Reingold and Tilford 1981) and its extension to trees with branch lengths (i.e., nonlayered, van der Ploeg 2014).

Here, we describe the algorithm behind this layout, illustrate its advantages for the visualization of phylogenetic trees using simulated and biological data (the measles virus phylogeny), and detail some of its features and limitations.

## New Approaches

We present here briefly the algorithm that allows obtaining the nonlayered tidy tree layout (proposed by van der Ploeg 2014), and we give illustrative examples of its use in the next section. We take as a starting point a tree plotted as in figure 1*B* (left). Nodes with no descendants are called tips. Nodes that are not tips have descendants that are their children. The node without an ancestor is called the root. All nodes are iteratively visited, from the rightmost one to the root, in decreasing order of their *x*-coordinates. For each node, we can define an “envelop,” that is, a polygon that exactly envelops the subtree associated with this node. The topmost segments of this envelop form the “top contour” of the subtree, and the bottommost segments form its “bottom contour.” For each node, the top contour of its bottom child is compared with the bottom contour of its top child. If the minimum distance between the two contours (red lines in fig. 1*A-*3) is larger than a predefined distance *d*, the top child is moved down until *d* is reached. If it is smaller, it is moved up. The current node is then moved along the *y*-axis to be centered on its children. At the end of this process, the tree is considered fully “compressed” (all red lines in fig. 1*B* have the same length, *d*). To take into account tip labels, that is, prevent labels from overlapping with other branches, their width is simply added to the corresponding terminal branch prior to tree compression. Any other information associated with each tip (sequence, synteny information, etc.) can be treated the same way.

For details on the algorithm and demonstration of its linearity, refer to the original article (van der Ploeg 2014).

## Illustrative Examples

In order to illustrate the gain in space and the impact on tree shape with this new layout, we applied it on two different phylogenies, a simulated one and a biological one (a phylogeny of virus).

First, a single tree with 713 leaves was randomly simulated under a birth–death process (speciation rate = 1, extinction rate = 0.9, stopping when reaching 100 extant taxa) and was plotted with two layouts: the classical phylogram layout (fig. 2*A*) and with the new nonlayered tidy tree layout (fig. 2*B*), keeping the same scale for the *y-*axis. We observe 1) that the level of compression is very high, that is, that the vertical space taken by the tree is drastically reduced, even though the minimum vertical distance between two contemporaneous branches is unchanged and 2) that the large vertical lines close to the root of the tree are also largely decreased, indicating that sister nodes (nodes having the same parental node) are getting much closer to each other along the *y-*axis.

Second, we retrieved the phylogeny of 79 measles virus genomes from the United States, using the NextStrain platform (Hadfield et al. 2018; Sagulenko et al. 2018). Again, we plotted this rooted phylogeny with the classical phylogram layout (fig. 3, left) and with the new layout presented here (fig. 3, right), but without forcing the *y*-axis to have the same scale. It appears with this new layout that the tips are more distinct (dots at each tip do not overlap anymore) and that the branches are less densely displayed. In this example, the level of compression is around 43%, that is, the tree takes 1.78 times less vertical space than with the classical layout. We observe that branches and nodes are less packed, which can be an advantage in cases where space is a limiting factor.

In addition to these two examples, we simulated an extra tree under a birth–death process (speciation rate = 1, extinction rate = 0.8, stopping when reaching 20 extant taxa), and we set up a public web page (https://damiendevienne.github.io/non-layered-tidy-trees/) to explore it. Two layouts are proposed, the classical phylogram layout and the tidy layout, and trees are interactive, that is, exploration of the tree is done by zooming and panning, and tip labels can either be set visible or invisible. This allows users to get a better insight into this new layout when associated with a zoomable interface. Under this setting, it appears that by bringing sister nodes closer to each other, the tidy layout allows visualizing more descendants of a given node at a given zoom level than with the classical layout (see screenshots of the interface in supplementary fig. S1, Supplementary Material online). This is especially true for deep nodes (nodes close to the root).

## Additional Notes Concerning the Tidy Layout

The level of compression with this layout depends on multiple factors. First, it depends on the number of tips and on their position along the *x* axis, relative to the root. To get an idea of this effect, we simulated phylogenetic trees with a birth–death process, and we varied the extinction rate between 0 and 0.8 (fixing speciation rate to 1; see supplementary material online). We observed that the level of compression went from 0% when the extinction rate was set to 0 (because the trees were ultrametric) to >50% when the rate was equal to 0.8 (supplementary fig. S2, Supplementary Material online). Second, the level of compression depends on the starting point, that is, how the tree is organized prior to tidying. Indeed, in any phylogenetic tree, subtrees associated with each node can be rotated without changing the relationship between the tips. It is likely that some representations are more “compressible” than others. Finding an algorithm that finds the representation that ensures the maximum possible compression would be an interesting follow-up of this work. Third, the level of compression depends on tips labels. Trees with very long tip labels or with extra information associated with tips are less compressible than trees without, unless a zoomable interface is used for visualizing the tree. In this case, one can zoom up to a point where the overlap between the tip label and the other branches disappears (see https://damiendevienne.github.io/non-layered-tidy-trees/).

The idea of tucking shorter branches underneath other branches in evolutionary trees is not new. Back to the second half of the 19th century, authors like Haeckel (1866) and Darwin (1859) drew famous trees that were based on this principle. While the advantage of this layout in terms of a gain in space is clear, and even though it can be helpful for better understanding relationships at deep nodes (supplementary fig. S1, Supplementary Material online), this representation can also have drawbacks. For instance, if the tree plotted is not a time tree (a phylogenetic tree scaled to time, Hedges and Kumar 2009), this layout may give the wrong impression that tips underneath others are extinct, which they are not. Also, by mixing tips along the *y-*axis, this layout can lead to a less clear distinction between monophyletic groups for some trees (compare the trees in fig. 3), which may not be desirable in some situations.

There are advantages and disadvantages to all layouts. The nonlayered tidy tree layout presented here is not meant to replace others. It is an additional option for researchers to explore their phylogenies of interest that will bring, in some cases, advantages over the other ones.

## Conclusion

Phylogenetic trees are present everywhere but can be hard to understand sometimes, especially when they contain many tips or when the space available to print them is limited. The layout we present here addresses this problem by allowing the trees to be printed in smaller spaces and by alleviating graphical issues inherent to other layouts, such as long vertical lines connecting sister nodes sometimes. We call for its adoption by all phylogenetic tree viewers such as Dendroscope (Huson et al. 2007), *ggtree* (Yu et al. 2017), *iTOL* (Letunic and Bork 2007), or *Figtree* (Rambaut 2018) to only cite a few.

## Supplementary Material

msac204_Supplementary_Data
