# Supplementary material for: tidy tree: A New Layout for Phylogenetic Trees
Source: Mol Biol Evol. 2022 Sep 26;39(10):msac204. doi: 10.1093/molbev/msac204 (PMC9550987; doi:10.1093/molbev/msac204)
Supplement: msac204_Supplementary_Data [file msac204_supplementary_data.pdf]

Supplementary material

Supplementary Method

To test the effect of the level of ultrametricity of the tree on the level of compression, we simulated trees with a birth-death process, using the *rphylo()* function in the ape package.

Speciation rate was set to 1 for all simulations. Extinction rate was varying between 0 and 0.8 (with steps of 0.2) and for each value of extinction rate, we simulated 100 trees, ending each time the simulation when the tree was reaching 100 taxa.

For each tree we compared the vertical space taken with the classical layout (y1) and with the new layout (y2) and computed the level of compression as a percentage:

$$comp = \frac{(y1-y2)}{y1} \times 100$$

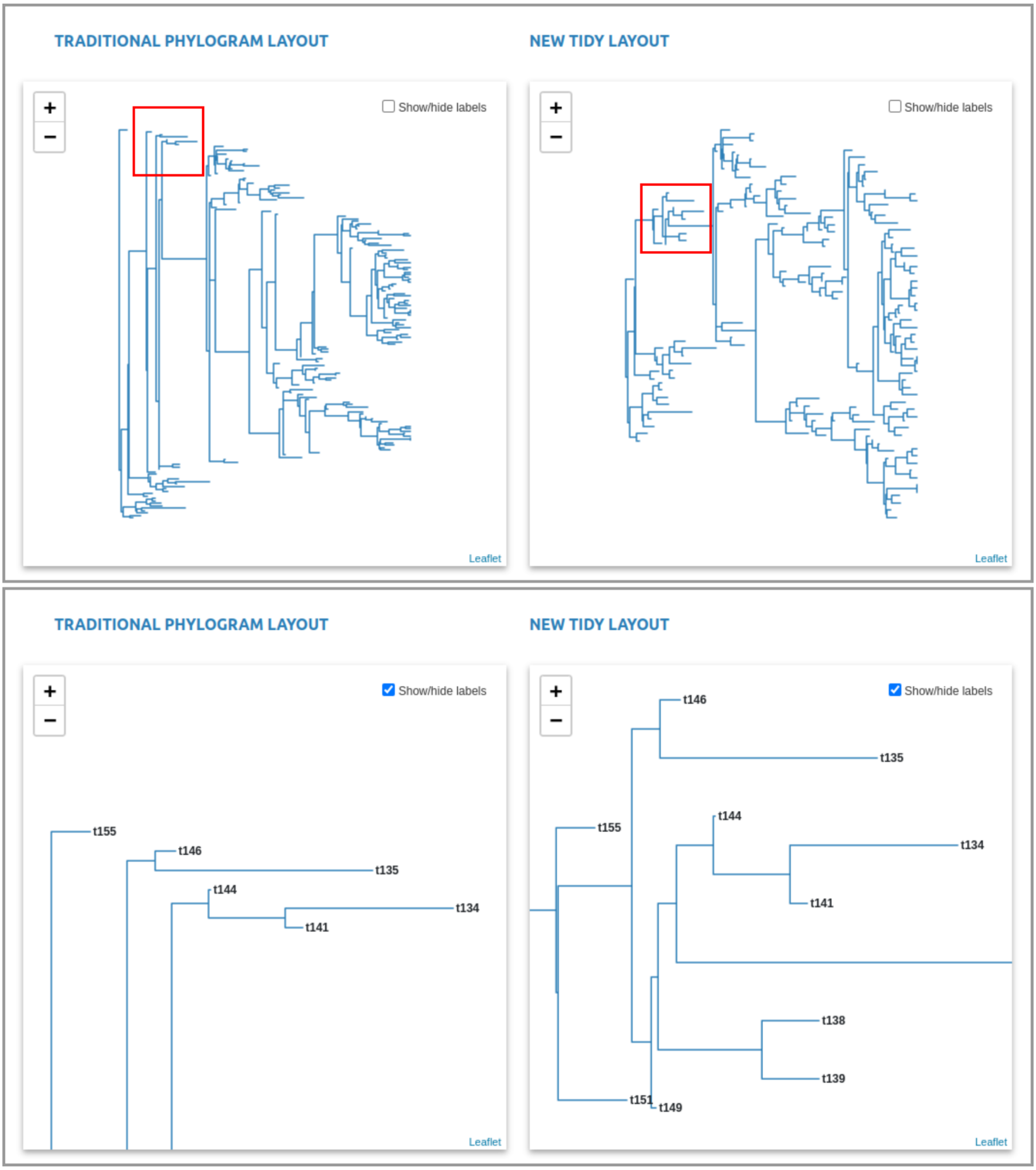

Figure S1: two screenshots of the web interface for comparing the classical phylogram layout (left tree on each panel) and the non-layered tidy tree layout (right tree on each panel) introduced in this work. The red squares on the top panel shows the portion of the tree on which the zoom is centred on the bottom panel. By bringing sister nodes closer to each other, the tidy layout allows for more nodes belonging to the same clade to be visible at a given zoom level, as compared to the classical layout. This web interface is accessible at https://damiendevienne.github.io/non-layered-tidy-trees/.

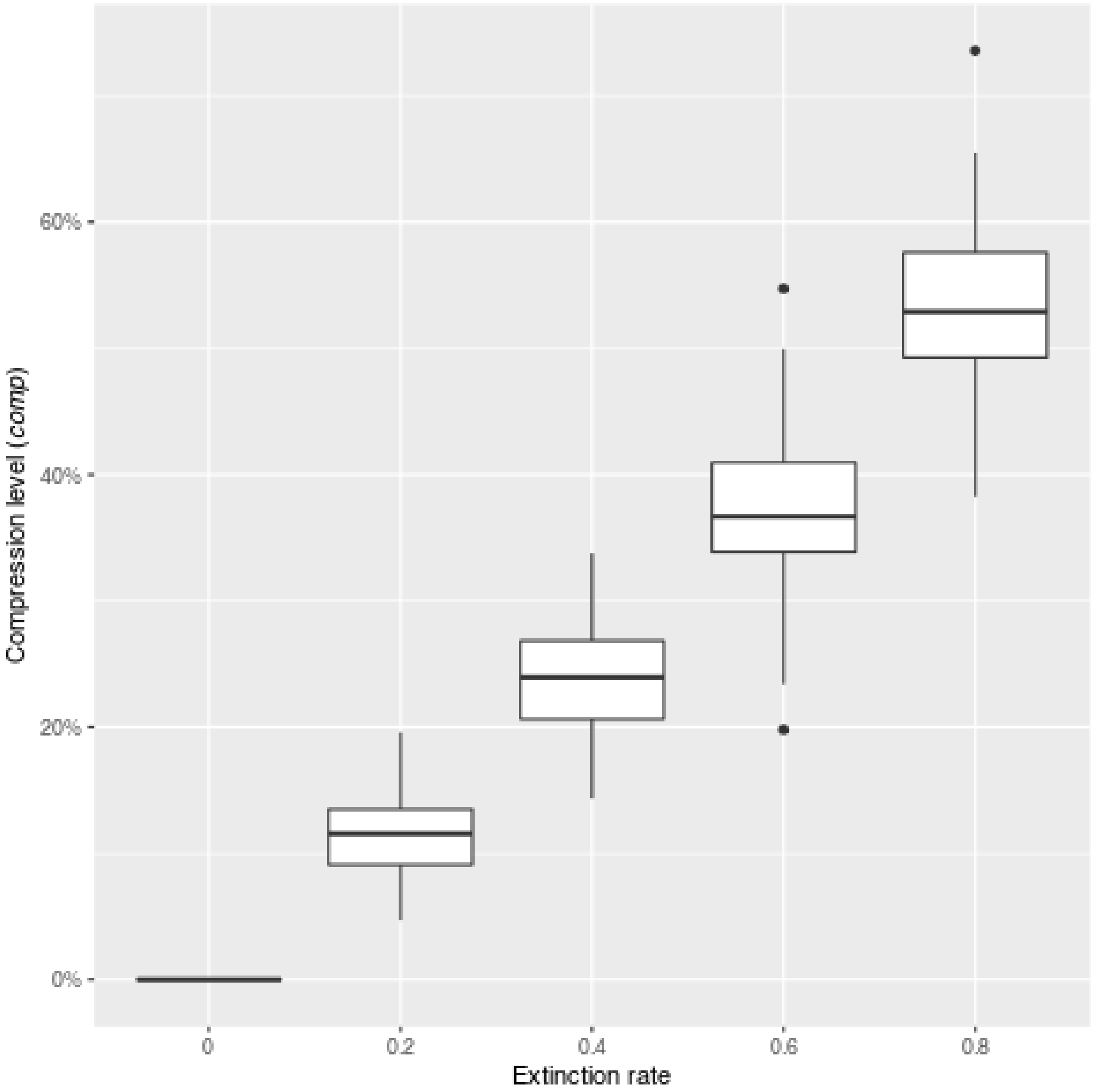

Figure S2: Relationship between the rate of extinction in the birth-death process generating phylogenetic trees and the level of compression achieved by the new non-layered tidy tree layout.
